# A randomized controlled study of immediate versus delayed umbilical cord clamping in infants born by elective caesarean section

**DOI:** 10.1186/s13052-020-00835-2

**Published:** 2020-05-24

**Authors:** Giuseppe De Bernardo, Maurizio Giordano, Rita De Santis, Paola Castelli, Desiree Sordino, Daniele Trevisanuto, Giuseppe Buonocore, Serafina Perrone

**Affiliations:** 1 Division of Pediatrics Neonatology and NICU, Ospedale Buon Consiglio Fatebenefratelli, Via Manzoni 220, 80123 Naples, Italy; 2 Department of Clinical Medicine and Surgery, Federico II University, Naples, Italy; 3 School of specialization in Pediatrics, Catholic University of the Sacred Heart Faculty of Medicine and Surgery, Rome, Italy; 4 School of specialization in Pediatrics, University of Pavia Faculty of Medicine and Surgery, Pavia, Italy; 5 Department of Emergency-NICU, A.O.R.N. Santobono-Pausilipon, Naples, Italy; 6 Department of Woman’s and Child’s Health, University of Padova, Padova, Italy; 7 Department of Molecular and Developmental Medicine, University of Siena, Siena, Italy; 8 Department of Medicine and Surgery, University of Parma, Parma, Italy

**Keywords:** Newborns, Delayed cord clamping, Elective caesarean section

## Abstract

**Background:**

Delayed umbilical cord clamping is associated with greater haemoglobin concentration and iron storage between 3 and 6 months of life and with less need of blood transfusion and lower incidence of neonatal hypotension compared to early umbilical cord clamping.

**Methods:**

The aim was to test the hypothesis that delayed cord clamping is better than early cord clamping in term infants born by elective caesarean section. Group A was subjected to immediate cord clamping while in the Group B, the umbilical cord was clamped 1 min after birth. Primary aim was revealed the difference in pre-ductal saturation between two groups while secondary aim was investigating the difference in HR, Ht, bilirubin and glycaemia. Pre-ductal SpO_2_ and HR were recorded at 5 and 10 min after birth, T was analysed 10 min after birth, glycaemia was revealed at 120 min while Ht and bilirubin were collected at 72 h.

**Results:**

132 newborns were enrolled in the study and allocated in ratio 1:1 to group A or B. Delayed cord clamping did not improve SpO_2,_ HR and T values compared to immediate cord clamping (*p* > 0,05). However, Group B showed greater haematocrit and bilirubin values at 72 h compared to Group A (56,71 ± 6663 vs 51,56 ± 6929; *p* < 0,05 and 8,54 ± 2,90 vs 7,06 ± 2,76; *p* < 0,05). Glycaemia value did not differ between two groups (*p* > 0,05).

**Conclusions:**

Group B did not reveal any differences in SpO_2_, HR, T and glycaemia compared to Group A. Group B showed greater values of haematocrit and bilirubin but without need of phototherapy.

**Trial registration:**

Umbilical Cord Clamping: What Are the Benefits; NCT03878602. Registered 18 March 2019 retrospectively registered.

## Background

Umbilical cord cutting determines the separation of the newborn from mother. Umbilical cord clumping consists in its binding by nipper to interrupt blood flow from placenta to foetus [1]. Experimental studies, executed on animals and humans, analysed cardiocirculatory changes in the foetus immediately after birth and the importance of the delayed cord clamping (DCC) for the hemodynamic stabilization, particularly in the lowest gestational age [2, 3]. In the eutocic delivery there are two modalities to obtain umbilical cord clamping: the first is immediate umbilical cord clamping (ICC) within 30s from birth, the second one is delayed DCC at least 1 min after birth because cerebral blood flow is reduced again due to lower cardiac output [4, 5]. DCC is better than ICC because it is associated with a great haemoglobin concentration in the newborns and best iron storage between 3 and 6 months of life and less incidence of transfusion and neonatal hypotension [6–9]. In a recent randomized study conducted in Nepal on 540 newborns, birth by eutocic delivery at term of gestational age, showed that DCC after 3 min of life is correlated with a better haemoglobin level and less incidence of anaemia at 8 months of life [10]. Association of Italian Hospital Gynaecologists Obstetricians declared contraindicated conditions to execute a DCC: Hypoxic-ischemic events: detachment of placenta, prolapse of the funiculus, uterine rupture, shoulder dystocia, premature rupture of foetal membranes, placenta previa, maternal collapse, embolism amniotic, maternal cardiac arrest, monochorionic twins, foetal hydrops, umbilical cord damaged, isoimmunization Rh. Zhou et al. conducted a meta-analysis to evaluate the differences between caesarean section (CS) and vaginal delivery (VD) with regard to hematologic parameters obtained by umbilical cord, placenta and newborns’ blood. It was revealed that CS was associated with an increased placental residual blood volume and a decreased level of iron-related haematological indices including haematocrit, haemoglobin, and erythrocyte in both cord and peripheral blood in term neonates at 3 or 6 h of life. This difference on placental transfusion could be caused to a weaker transfusion force and a shorter transfusion period during CS due to ICC. Weighted mean difference of haematocrit value was significantly greater in infants born by elective caesarean section (ECS) than those born by emergency CS [11]. However, authors did not analyse the effects of the timing of cord clamping on hematologic parameters in relation to the different mode of delivery. Therefore, despite the evidence of beneficial effects for umbilical cord delay after eutocic delivery, this practice has not yet been evaluated after ECS.

## Material and methods

### Aims

Primary aim of the study was evaluating the effect of DCC compared to ICC on pre-ductal SpO_2_ in infants born by ECS. Secondary aims were analysing the differences of heart rate (HR), temperature (T), glycaemia, haematocrit (Ht) and bilirubin.

### Participants

Non-commercial study, case-control, randomized, open was conducted to Department of mother and child’s health, Poliambulanza Foundation, Brescia. The study was recorded and publicly accessible at https://clinicaltrials.gov/ct2/show/NCT03878602?term=de+bernardo&rank=3. Term infants born by ECS were enrolled between March and August 2018 and assigned to Group A if were subjected to ICC and to Group B if were subjected to DCC. The study was approved by Brescia Ethical Committee and written informed consent was obtained from the families. Eligibility criteria for mothers were BMI > 19 and < 25 and age ≤ 37 years. They were excluded mothers with pathologies, toxicomaniac and those who smoked or assumed drugs during pregnancy. Eligibility criteria for newborns were, gestational age = 37–42 wks, birth body weight appropriate for gestational age. Newborns admitted in NICU, and who needed neonatal resuscitation were excluded. They were excluded also newborns that showed hypoxic-ischemic events: detachment of placenta, prolapse of the funiculus, uterine rupture, shoulder dystocia, premature rupture of foetal membranes, placenta previa, maternal collapse, embolism amniotic, maternal cardiac arrest. Finally, they were excluded monochorionic twins, foetal hydrops, umbilical cord damaged, isoimmunization Rh, respiratory and malformative diseases.

### Procedure and randomization

Umbilical cord management was established before birth. All eligible infants were assigned to group A or B by block randomization thanks a statistical software. In operating room, a nurse turned on a timer after birth and gynaecologist clamped umbilical cord immediately after birth or at 1 min. DCC was chosen at 1 min to avoid maternal bleeding, infections or other surgery-related complications [11]. It was promoted neonatal bonding to improve mother-child relationship and then the baby was covered with sterile towels. After neonatal bonding, the infant was positioned under the infant warmer, dried and stimulated. A pulse-oximeter (Covidien) was utilized to evaluate newborn saturation level and positioning the probe on the right hand or wrist, reference values were 80–85% and 85–95%, respectively at 5 min and 10 min after birth [12]. HR was evaluated by a Cardiomonitor (Transport Pro, GE Healthcare) and positioning the probes on the chest of the newborn, reference values were 100–150 bpm [12]. T was analysed by the probe of the infant warmer (Panda Warmer, GE Health Care). Pre-ductal saturation and HR were recorded at 5 and 10 min after birth while T was analysed at 10 min after birth along with Apgar Score to evaluate perinatal adaptation of the newborns. Capillary blood samples of the heel were analysed by ABL90 Flex to evaluate glycaemia (at 120 min from birth), Ht and bilirubin (at 72 h from birth). These variables were collected in according to protocol procedures to monitor neonatal health status. During hospital stay was promoted rooming-in 24 h. Data were recorded using a database (Excel 2007) by a nurse that was aware of the study aims. Parameters collected were sex, gestational age, birth body weight, Apgar score at 5 and 10 min, HR, pre-ductal SpO_2_, T, glycaemia, Ht, bilirubin.

### Statistical analysis

Statistical analysis was carried out by a statistician who was aware of the study aim using SPSS version 25.0 for Windows (IBM, Armonk, NY, USA). Normal distribution of data was evaluated by Kolmogorov-Smirnov test. Parametric data were analysed by ANOVA repeated measures between-whitin or ANOVA, as appropriate. Non-parametric data were analysed by χ^2^ test. Sample size required to execute this study was 132. This analysis was computed by G*Power 3.9.1.2 for windows [13] setting: α = 5%, β = 10%, number of measurements = 2, correlation of repeated measures = 0.5, non-sphericity correlation = 1, effect size = 0,14. In 1969 Cohen has defined the following conventional effect sizes if data was not available to calculate it without overestimating the power: small f = 0.14; medium f = 0.25; large f = 0.40 [14]. All data with *p* < 0,05 were considered statistically significant.

## Results

**Fig. 1:**
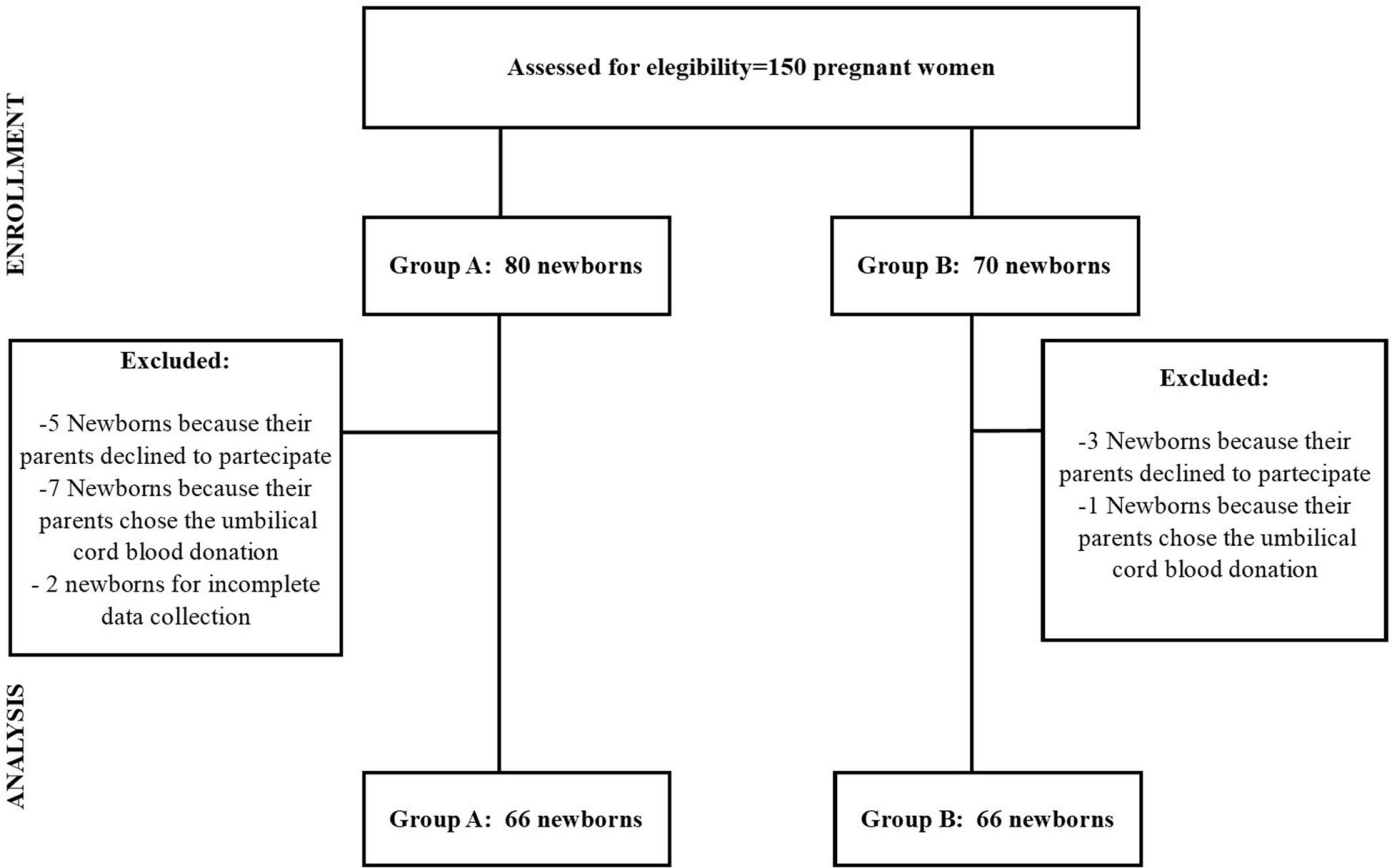
Flow diagram of the study population from assessment for eligibility to analysis

A total of 150 pregnant women were approached for recruitment. However, in the final analysis were included 132 term newborns and allocated in ratio 1:1 in group A or B (Fig. 1). There were not statistically significant differences between groups for SpO_2,_ HR and T both at 5 min and at 10 min from birth (Table 1). No difference was also found in glycaemia at 120 min, but greater values Ht and bilirubin were observed in group B compared to group A at 72 h of life (Table 1).

**Table 1:** Change in vital parameters, glycaemia, haematocrit and bilirubin in newborns subjected to DCC or ICC

Outcome		Time	N	Group A	Group B	F (1,127)	P_value	R-value
**SpO**_**2**_	**T0 vs T1**	**5 min**	132	88,98 ± 7027	89,169 ± 7453	0,362	0,548	0,089
		**10 min**	132	95,328 ± 3976	96,046 ± 3346			
**HR**	**T0 vs T1**	**5 min**	132	164 ± 18,191	157,89 ± 15,66	0,233	0,630	0,07
		**10 min**	132	163 ± 15,313	155,86 ± 13,571			
**T (°C)**		**5 min**	132	36,60 ± 0,642	36,64 ± 0,640	–	0,745	–
**Glycaemia (mg/dl)**		**120 min**	132	60,32 ± 9937	56,02 ± 17,101	–	0,080	–
**Ht (%)**		**72 h**	132	51,56 ± 6929	56,71 ± 6663	–	0,001*	**–**
**Bilirubin (mg/dl)**		**72 h**	132	7,06 ± 2,76	8,54 ± 2,90	–	0,004*	**–**

Notes: Data expressed as mean ± SD; N: number, HR: heart rate; *p < 0,05

## Discussion

Our findings indicated that delaying cord clamping after 60 s increased haematocrit after 72 h of life born by ECS, without influencing the SpO_2_, HR, T and phototherapy. The placental transfusion by DCC gives to baby about 80–100 ml of blood additional and 20–30 mg of iron [15], this determines a great haemoglobin concentration in the newborns and best iron storage between 3 and 6 months of life and less incidence for transfusion and neonatal hypotension [6–9, 16]. Experimental studies, executed on animals and humans, analysed cardiocirculatory changes in the foetus immediately after birth and the importance of the DCC for the hemodynamic stabilization, particularly in the lowest gestational age Delayed cord clamping alters acid-base parameters and lactate values compared to immediate cord clamping [2, 3].. The timing of cord clamping was 180 s or longer demonstrating neurodevelopmental benefits in low-risk populations [17, 18]. DCC after one minute is a practice that has been shown to be beneficial in spontaneous births [19–21]. The infants born by ECS showed a lower value of red blood cells than those birth by VD. The factors that affect placental transfusion appear to be uterine contractility. CS reduces placental transfusion due to maternal hypotension and insufficient uterine contractions [11–22]. Such reduction is even more pronounced by ECS than in emergency CS [11]. No differences in maternal bleeding complications were found with DCC in multiple pregnancies compared to ICC. DCC can be done safely in multiple pregnancies without any increased of maternal risk [23]. DCC is not possible without the parents’ informed consent because this practice can alter the procedure to donation and collection of umbilical cord blood. Indeed, umbilical cord must be clamped and severed immediately to proceed with collection successfully. Our study evaluated whether the benefits that the DCC presented in the spontaneous births occur also for those born at term by ECS. Our data suggested that DCC was associated with an increase in haematocrit and bilirubin estimated at 72 h after birth. Although DCC was associated in a study [24] with an increase in phototherapy, in our study capillary bilirubin values were higher in the DCC group compared to the ICC group but without the need for phototherapy. Furthermore, no difference in statistical significance was found in HR and SpO_2_ between groups. These data were recorded in the first and 5th minute from birth. Wafaa at al. found greater values of SpO_2_ between newborns subjected to DCC compared to those treated by ICC, immediately after the birth. However, at 6 h of life the difference was no longer found [4]. Yu L et al. did not consider the evaluation of SpO_2_ and HR in DCC group but considered as primary outcomes mortality, risk of iron-deficiency anaemia [20]. Similarly, Nevill at al. did not analyse oxygen saturation at birth but assessed the need for oxygen support which resulted lower in the group with DCC compared to ICC [9]. A large trial on 1510 newborns born by VD and randomized in DCC or ICC group reported higher values of SpO2 and lower value of HR at 1 and 5 min in DCC group with respect to IC [25]. These results encourage the use of DCC also in the newborns born by ECS as a valid tool to obtain a smoother cardiopulmonary transition. In our study although no statistical difference was found in HR and SpO2 although infants in the DCC group showed higher pre-ductal saturation values and lower HR values than those in the ICC group. This means that it is probably necessary to conduct a study in a larger sample size of newborns born by ECS to reveal a statistical difference between these variables. Finally, the temperature was evaluated for the potential heat loss during DCC. Data showed no clinically relevant temperature difference at the time of admission to the nursery between two arms, according to a recent systematic review [26]. The temperature variable was assessed in 11 trials involving 2317 preterm infants. Although there was moderate heterogeneity between studies a reduction of temperature in the group with DCC was not observed [26]. The strengths of our study included study design and attention for newborns born by ECS, however we investigated only the short-term outcome. It could be interesting to carry out studies about long-term outcomes such as iron concentration and neurodevelopment in childhood.

## Conclusion

DCC in patients born by ECS is a valid practice and can be performed. DCC group improved HT blood values while did not influence HR, SpO_2_, T and glycaemia compared with ICC group. The lack of any difference in neonatal body temperature between the two modalities of clamping confirmed the safety of DCC also in this patient population. There was an increase in bilirubin in newborns born by DCC but without the need phototherapy. All together these results strongly support the hypothesis that DCC is better than ICC in term infants born by ECS.

## Data Availability

The datasets generated and/or analysed during the current study are not publicly available due privacy reasons but are available from the corresponding author on reasonable request.

## Abbreviations

ICC: immediate umbilical cord clamping
DCC: delayed umbilical cord clamping
CS: caesarean section
VD: vaginal delivery
ECS: elective caesarean section
HR: heart rate
T: Temperature
